# Unruptured Multiple Sinus of Valsalva Aneurysms

**DOI:** 10.1155/2020/5046095

**Published:** 2020-05-27

**Authors:** Demian J. Omeh, Amgad N. Makaryus

**Affiliations:** ^1^Department of Medicine, Nassau University Medical Center, East Meadow, NY, USA; ^2^Department of Cardiology, Nassau University Medical Center, East Meadow, NY, USA; ^3^Donald and Barbara Zucker School of Medicine at Hofstra/Northwell Health, Hempstead, NY, USA

## Abstract

Sinus of Valsalva aneurysm (SVA) is a rare cardiac condition occurring in about 0.09% of the general population, with potential for grave complications. Unruptured sinus of Valsalva aneurysms of all three sinuses in the same patient are even more rare. There are congenital, inherited, or acquired causes. Noninvasive cross-sectional imaging modalities, consisting of color Doppler echocardiography, cardiac computed tomographic angiography (CCTA), and cardiac magnetic resonance imaging (MRI), make the diagnosis. Treatment is mainly by open surgical reconstruction. However, transcatheter techniques are gaining popularity with noninferior outcomes in selected cases. We report the diagnosis and successful management of a patient with an unusual presentation of multiple unruptured SVAs of all three sinuses, and we conducted a review of the English medical literature.

## 1. Introduction

Sinus of Valsalva aneurysm (SVA) is a rare cardiac condition that is well recognized for its devastating complications when rupture occurs. The sinuses of Valsalva (SV) are dilitations of the aortic wall located between the aortic valve and the sinotubular junction. The nomenclature of SV is related to the coronary arteries, designated as the right coronary sinus (RCS), left coronary sinus (LCS), and noncoronary sinus (NCS). SVA is the abnormal dilation of the aortic sinus above the supra-aortic ridge resulting from the separation of the aortic media from the annulus fibrosis. SVA can be congenital, inherited, or acquired. Acquired SVAs can have infectious, degenerative, traumatic, or senile etiology. In a large autopsy series, the incidence of SVA was reported as 0.09% in the general population [1]. The aneurysmal malformation usually involves a single cusp. The majority of SVAs originate from the right sinus of Valsalva and account for 65-85% of cases [2, 3]. The SVAs arising from the noncoronary sinus account for 10-30% [2, 3]. SVAs from the left sinus of Valsalva are exceedingly rare and account for less than 5% [2, 3]. Unruptured multiple sinus of Valsalva aneurysms in the same patient are extremely rare, and only very few cases of unruptured multiple aneurysms of all three sinuses have been documented [4]. We report the diagnosis and successful management of a patient with an unusual presentation of multiple unruptured SVAs of all three sinuses.

## 2. Case Summary

**Figure 1:**
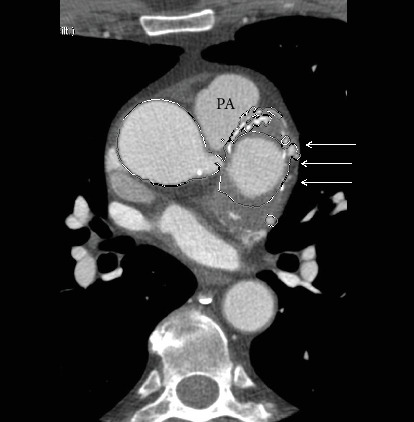
Axial CT image showing the large left SVA causing pulmonary artery (PA) displacement and showing internal calcification and thrombosis (arrows).

**Figure 2:**
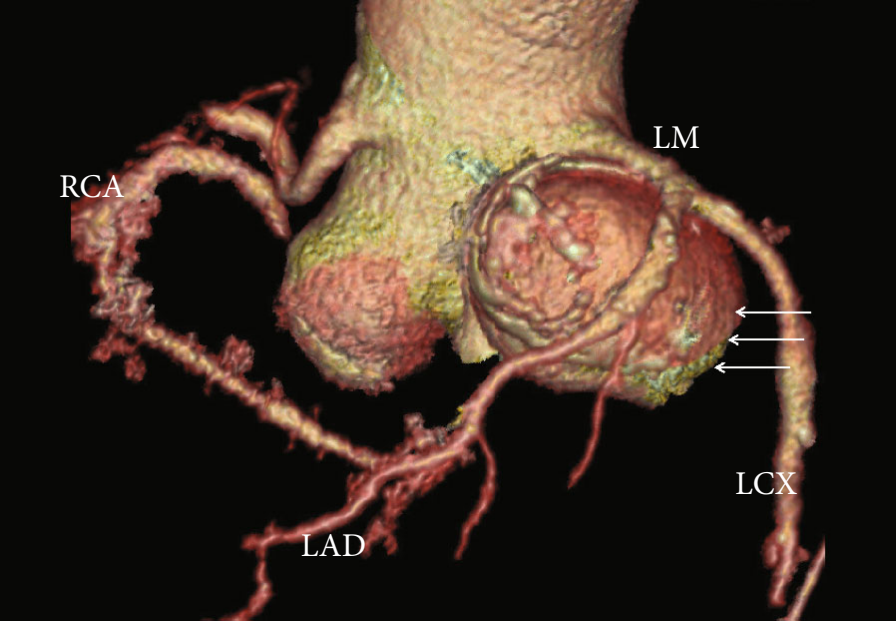
Three-dimensional volume-rendered image of the aortic root showing the large left SVA (arrows) and the right SVA (LM = left main coronary artery; LAD = left anterior descending artery; LCX = left circumflex artery; RCA = right coronary artery).

A 63-year-old man presented with nonproductive cough for a few months. Medical history was significant for hypertension. He had a remote 20 pack-year smoking history but had quit 25 years prior. He reported a family history of abdominal aortic aneurysm. On physical exam, the heart rate was 80 beats/min, and the blood pressure was 148/80 mmHg with an oxygen saturation of 99% on room air. There was no carotid bruit, and the cardiac examination revealed regular rate and rhythm and normal S1 and S2 without any murmur, rub, or gallop. The rest of the physical exam was unremarkable. Cardiac CT angiography (CCTA) with reconstruction of the aortic root was obtained to assess for possible causes to the patient's presentation (Figure 1). The CCTA showed a large left calcified SVA measuring 6.3 cm in maximal diameter with proximal extension to the aortic valve annulus and distal extension to the left coronary main stem causing pulmonary artery displacement. A 2.3 cm right SVA and a 1.8 cm noncoronary SVA were also noted (Figure 2). The left circumflex artery showed a calcified plaque in the proximal portion causing mild luminal narrowing. The left anterior descending artery and right coronary artery were without plaque. The patient underwent surgical resection of the aneurysms, including a complete root replacement with coronary implantation and aortic valve replacement. The aortic valve was replaced with a 25 mm bovine pericardial composite valve graft, and the coronary arteries were reimplanted. A large thrombus in the left aneurysm was successfully evacuated. The patient had an uneventful postoperative recovery and did well.

## 3. Discussion

Sinus of Valsalva aneurysm is a rare cardiac defect that is well recognized for its most feared complication: rupture with aortocardiac or aortopericardial shunt and profound hemodynamic instability. Based on a large autopsy series of over 8,000 individuals, the incidence of SVAs in the general population is 0.09%. In open-heart surgery cases, the incidence is 0.14–0.96%. Congenital SVAs make up to 0.1% of all congenital heart defects. Incidence as high as 3-4.5% has been reported in some Asian populations. It has a male predominance with a male to female ratio of 4 : 1 [1, 3, 5].

SVAs can be congenital, inherited, or acquired. The congenital SVAs are consequences of incomplete fusion of the distal bulbar septum (primitive bulbus cordis) and truncal ridges (aortopulmonary septum) resulting in fragility at the junction of the aortic annulus, the right aortic sinus media, and the right portion of the noncoronary sinus. They are commonly associated with ventricular septal defects (30–50%), aortic regurgitation (20–30%), bicuspid aortic stenosis (10%), and less commonly, atrial septal defect, pulmonic stenosis, coarctation of the aorta, subaortic stenosis, tetralogy of Fallot, or patent foramen ovale [3]. Inherited forms occur in persons with deficiency of healthy elastic tissue resulting in annuloaortic ectasia with dilatation of all three SVs and thereby progressive effacement of the sinotubular junction as seen in Marfan, Ehlers-Danlos, and Loeys-Dietz syndromes. Acquired aneurysms can result from atherosclerosis, infective endocarditis, tuberculosis, syphilis, dissecting aortic aneurysms, cystic medial necrosis, Behcet's disease, and traumatic injury involving the aortic root [3]. Our patient had atherosclerosis as a risk factor.

The aortic root represents the portion of the aorta and left ventricular outflow tract demarcated by the sinotubular junction superiorly and the basal parts of the aortic valve leaflets inferiorly. Thus, the aortic root is made of the aortic valve leaflets, the commissures, the interleaflet triangles, the SVs, the sinotubular junction, and the annulus [6]. The Sinuses of Valsalva play an essential role in aortic valve function. They provide a space to prevent blocking of the coronary artery orifices from the open aortic leaflets. Secondly, they favor the development of eddy currents behind the open leaflets, which in turn enhances the prompt closure of the aortic valve leaflets at the end of systole [3, 7].

Most unruptured SVAs are asymptomatic and detected as incidental findings on imaging studies. Most of the patients become symptomatic in the fourth and fifth decades [8]. Large unruptured SVAs can compress the adjacent cardiac structures resulting in right ventricular outflow obstruction, aortic insufficiency, tricuspid stenosis, tricuspid insufficiency, mitral insufficiency, ischemia/infarction, infective endocarditis, thromboembolism, mass effect, and conduction disturbance. Our patient presented with chronic cough and a mediastinal mass with a displacement of the pulmonary artery. Congenital and inherited forms usually manifest with a rupture in the third or fourth decade of life. Patients with ruptured SVA present with continuous systolic murmur and features of acute decompensated heart failure [9]. Multiple aneurysms are uncommon, and multiple unruptured aneurysms, as in our patient, are extremely rare.

Chest radiograph findings are dependent on SVA location, size, and presence or absence of rupture. Patients can have an abnormal cardiomediastinal silhouette and increased or decreased pulmonary vascularity [10]. However, chest radiograph findings are nonspecific and of low diagnostic significance. Traditionally, transthoracic echocardiography is the first screening modality of choice given its excellent sensitivity, availability, and portability. Many studies have reported >90% accuracy of echocardiography for detecting the SVA. Transesophageal echocardiography, due to its better acoustic window and higher resolution, provides a more precise characterization of the aneurysm [3, 11, 12].

Multislice CCTA provides a detailed anatomic depiction of SVAs and the surrounding cardiac structures. High-resolution 3D reconstructed images of CCTA (Figure 2) are invaluable in surgical planning. The disadvantage of CT is the use of ionizing radiation. Cardiac magnetic resonance imaging (MRI) can further evaluate the left ventricular hemodynamic pattern, identify aortic regurgitation, and quantify aortocardiac shunt or fistulous blood flow but may be unsuitable in the setting of acute SVA rupture due to its prolonged acquisition time. Invasive angiography has been considered the gold standard and has the advantage of both diagnostic and therapeutic potentials. However, noninvasive cross-sectional imaging modalities, consisting of color Doppler echocardiography, CCTA, and cardiac MRI, have mostly replaced it [3, 13].

There is no established specific guideline for the management of SVA. It is acceptable to follow the 2010 American Guidelines for the Diagnosis and Management of Thoracic Aortic Root Aneurysm: surgical repair is indicated in (1) aortic root diameter > 5.5 cm, (2) aortic root diameter > 5 cm in patients with bicuspid valves, (3) aortic root diameter > 4.5 cm in the setting of connective tissue disorders, and (4) a growth rate of >0.5 cm/year [14]. Ruptured and symptomatic unruptured SVAs will require surgical repair. The rest of the patients not meeting these criteria are managed medically with tight blood pressure control with angiotensin receptor blockers and *β*-blockers. Small symptomatic or ruptured SVA is repaired by primary closure. Exclusion of the aneurysmal sac, followed by patch closure using bovine pericardium, polyethylene terephthalate, glutaraldehyde-treated autologous pericardium, or polytetrafluoroethylene, is the preferred surgical technique for the repair of larger SVAs. Many recent reports have documented good clinical outcomes using transcatheter closure devices as surgical alternatives [4, 15]. In our patient, open surgical resection of the left sinus aneurysm and complete root replacement with coronary reimplantation and aortic valve replacement was undertaken given the size of the left sinus aneurysm.

Our case exhibits a rare combination of SVAs of all three sinuses with a displacement of the pulmonary artery by the 6.3 cm left aneurysm in a relatively asymptomatic patient. Although there was extensive aortic annulus and left main coronary stem aneurysmal extension, our patient was successfully managed surgically due to recognition and identification prior to aneurysm rupture.
